# Kefir: A Potential Gut Microbiota Modulator: A Systematic Review of Human Interventional Studies

**DOI:** 10.1002/mbo3.70297

**Published:** 2026-04-26

**Authors:** Mohammed Hamsho, Yazan Ranneh, Batoul Kaddour, Sundus Alfakhri, Hale Hacıbayram, Wijdan Shkorfu, Merve Terzi, Abdulmannan Fadel

**Affiliations:** ^1^ Department of Nutrition and Dietetics Faculty of Health Science, Istanbul Yeni Yuzyil University Istanbul Turkey; ^2^ Department of Nutrition and Dietetics College of Pharmacy, Al‐Ain University Abu Dhabi United Arab Emirates; ^3^ Department of Nutrition and Dietetics Faculty of Health Science, Istanbul Gelisim University Istanbul Turkey; ^4^ Department of Nutrition and Dietetics Faculty of Health Sciences, Bahcesehir University Istanbul Turkey; ^5^ Department of Nutrition and Health College of Medicine and Health Sciences, United Arab Emirates Al Ain United Arab Emirates

**Keywords:** colonization, fermented milk, gut microbiota, kefir, probiotic, systematic review

## Abstract

Kefir is a fermented dairy product containing live and active microbial culture, including lactic acid bacteria and yeast. Preclinical studies and narrative reviews have reported potential modulatory effects of kefir on gut microbiota composition. Despite this, there isn't yet a thorough analysis of human intervention studies that fills this gap of research. Therefore, the aim of this review is to examine the role of kefir consumption on gut microbiota composition in humans. Comprehensive research was conducted using three major databases including Web of Science, PubMed, and Scopus. The risk of bias was assessed using Cochrane risk‐of‐bias tool and Risk of bias of non‐randomized trials. The search resulted in 2743 studies, of which eight studies met our eligibility criteria. Overall, kefir resulted in minor changes in phyla and class levels. On the other hand, the *Bifidobacterium* genus increased in 3 out of 4 studies. Kefir‐specific bacterial species and strains were found in participants fecal samples suggesting colonization properties. Kefir consumption was associated with modest and heterogenous changes in gut microbiota composition. Proposed mechanisms include transient persistent of kefir‐associated taxa and modulation of the intestinal environment, although direct functional evidence in humans remains limited.

## Graphical abstract

Daily cow's milk kefir intake transiently enriches gut microbiota, modulates the intestinal environment by increasing SCFA and reducing pH and oxygen, and exerts antimicrobial effects via SCFAs, bacteriocins, exopolysaccharides, and microbial fractions, highlighting its potential for gut health promotion.

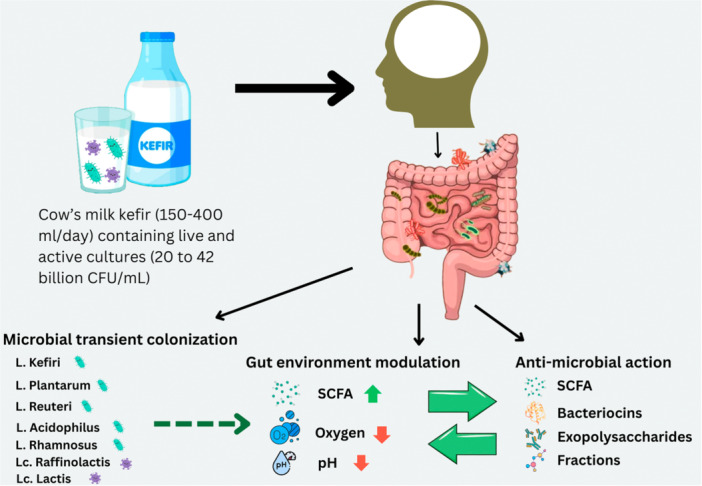

**Abbreviations**
16S rRNA: 16S ribosomal RNA
FAO: Food and Agriculture Organization
GMWI: the gut microbiome wellness index
IBD: inflammatory bowel disease
ICU: intensive care unit
ISAPP: International Scientific Association for Probiotics and Prebiotics
LAB: lactic acid producing bacteria
Qpcr: quantitative real time polymerase chain reaction
RCT: randomized controlled trial
ROBINS‐I: risk of bias of non‐randomized trials
ROB 2: risk‐of‐bias tool for randomized trials

## 1 Introduction

The human gut microbiota, a highly diverse community of bacteria, archaea, viruses, and fungi inhabiting the gastrointestinal tract, plays a critical role in maintaining host health. This complex ecosystem contributes to digestion, vitamin synthesis, immune regulation, and protection against pathogens (Peluzio et al. 2021). According to Food and Agriculture Organization (FAO), dysbiosis is defined as the loss of healthy microbiome which is characterized by reduction of microbiome diversity and shifts in the composition of the microorganism's population, resulting in impaired host physiological functions and development of chronic diseases such as metabolic syndrome (Alagiakrishnan et al. 2024). Because of these associations, the modulation of gut microbiota has become a central focus in preventive and therapeutic nutrition research (Ross 2024).

The establishment of gut microbiota begins early in life and is influenced by several factors, such as birth mode (vaginal *vs.* cesarean), infant feeding practices, antibiotics exposure, genetics, lifestyle, and diet (Kim and Jazwinski 2018). Among these, dietary intake is considered the most potent and modifiable determinant of gut microbial composition and metabolic output. Dietary components can selectively promote beneficial microorganisms or suppress potential pathogens, thereby shaping microbial balance and health outcomes (Ross 2024; Singh 2017).

Recently, the consumption of fermented food has received increasing attention as a nutritional strategy to beneficially modulate the gut microbiota (Leeuwendaal et al. 2022; Le Roy 2022). According to the International Scientific Association for Probiotics and Prebiotics (ISAPP), probiotics are defined as live microorganisms that, when administrated in adequate amounts, confer health benefits on the host (Ontario 2002; Hill et al. 2014). Similarly, functional foods are those that provide health benefits beyond basic nutrition, often through bioactive components that improve physiological functions or reduce disease risk (Vettorazzi et al. 2020; Temple 2022). Fermented milk products such as yogurt and Kefir are commonly categorized as functional foods due to their probiotics and bioactive profiles (Peluzio et al. 2021).

Kefir is a traditional fermented diary beverage produced through the symbiotic fermentation of milk using kefir grains which is a complex of matrix proteins and polysaccharides (mainly kefiran) that harbor a diverse consortium of bacteria and yeasts. The microbial composition of kefir typically includes species of *Lactococcus, Leuconostoc, Acetobacter*, and yeasts such as *Kluyveromyces marxianus* and *Saccharomyces cerevisiae* (Bell et al. 2018; Hamsho 2025). This complex microbial community interacts synergistically to produce metabolites such as organic acids, bacteriocins, and exopolysaccharides that may influence gut microbial ecology and host physiology (Anjana and Tiwari 2022).

Preclinical studies have provided compelling evidence that kefir supplementation could exert modulatory effects on gut microbial ecosystem by enriching health promoting taxa such as *Bifidobacterium* and *Akkermansia*, while concurrently suppressing potentially pathogenic or opportunistic microorganisms. In experimental models, kefir‐induced modifications in gut microbial composition have been associated with enhanced intestinal integrity, increased production of short‐chain fatty acids, and attenuation of mucosal inflammation through immunoregulatory mechanisms (Zhang et al. 2016). Taken together, preclinical and mechanistic evidence indicates that kefir may act as a complex, multifunctional probiotic system capable of modulating microbial community dynamics, restoring intestinal homeostasis, and ultimately supporting host gastrointestinal health (Peluzio et al. 2021).

However, clinical evidence from human intervention trials remains limited and inconsistent. Variability in study design including difference in kefir microbial composition, fermentation substrates, dosages, intervention duration, and sequencing methodologies, has contributed to heterogenous outcomes across studies. Methodological heterogeneity across existing trials substantially constrains the comparability of findings and impedes the establishment of a clear, evidence‐based understanding of kefir specific influence on gut microbiota diversity, structural composition and function.

Given the growing scientific and public interest in kefir as a functional food, there is an increasing need for a rigorous, evidence‐based synthesis of its effect on the human gut microbiota. Therefore, the objective of this systematic review is to (A) thoroughly identify and apprise evidence from human intervention studies examining the effect of kefir consumption on gut microbiota composition and (B) discuss the potential mechanisms proposed to underlie the observed changes.

## 2 Methods

We performed a systematic review based on the guidelines of the Preferred Reporting Items for Systematic Reviews and Meta‐Analyses (PRISMA) (Moher 2009). The study protocol was registered in PROSPERO with the following ID: CRD420251002110.

### 2.1 Search Strategy

A search of online databases including PubMed, Web of Science, and Scopus was conducted up to the cut‐off date of the fourth of March 2025 by two independent authors (H.H. and W.S.). The search was repeated on the Second of November 2025. We systematically searched the literature to identify human interventional studies that assessed the impact of kefir on gut microbiota. A comprehensive search was carried out to identify relevant studies by examining electronic databases using relevant Medical Subject Headings (MeSH) (Supporting Information S1: Table S1) and predetermined criteria. In addition, we manually checked the reference lists of included articles and relevant reviews to identify any additional studies meeting the inclusion criteria.

### 2.2 Eligibility Criteria

In this systematic review, studies were included if they met the following criteria: (1) utilized human interventional designs, including randomized controlled trials (RCTs), non‐randomized controlled trials, or cross‐over clinical trials; (2) involved administration of kefir exclusively derived from cow's milk, without any additional bioactive fortification (e.g., prebiotics, probiotics, synbiotics, herbs, or pharmaceuticals); (3) evaluated gut microbiota as a primary or secondary outcome using validated molecular‐based, culture‐independent techniques, such as 16S rRNA gene sequencing, shotgun metagenomic sequencing, or quantitative real‐time polymerase chain reaction (qPCR); (4) reported specific gut microbiota‐related outcomes, including changes in microbial diversity indices (e.g., alpha‐diversity metrics such as Shannon, Simpson, or Chao1 indices; beta‐diversity analyses such as Bray‐Curtis dissimilarity or UniFrac distance), relative abundance of bacterial taxa (at phylum, class, family, genus, species, or strain levels), or post‐intervention detection of kefir associated microbial species or strains in participants’ fecal samples; and (5) were peer‐reviewed, published as full‐text articles in the English language.

Conversely, studies were excluded if they were observational, case reports, in vitro or animal studies, literature reviews, meta‐analyses, conference abstracts, letters, or editorials; if the intervention involved kefir from non‐bovine sources, fortified kefir products, or co‐administration with other fermented foods; or if they assessed gut microbiota composition solely using culture‐dependent methodologies or failed to provide quantitative microbiota‐related outcome data.

### 2.3 Data Extraction

After the selection process of articles, with regard to the inclusion and exclusion criteria, characteristics of included interventional studies information including (study, country, study sample size (female and male participants), age (range), population health condition, kefir dose, kefir preparation methods, microbial content of kefir (strains, CFU), nutritional composition of kefir, and diet description) (Table 1); Gut microbiota outcomes following kefir intervention including (study, analysis method, taxonomic changes (Phylum/Class/Genus/Species), alpha diversity, beta diversity, and kefir strains colonization) (Table 2) were extracted by three independent authors (B.K., S.A. and M.T.) and confirmed by a third author (M.H.).

**Table 1:** Characteristics of included interventional studies.

Study	Country	Design	Duration	Sample size: F: (n), M (n)	Age (Range)	Health Condition	Kefir Dose	Preparation Method	Microbial content of kefir (Strains, CFU)
Walsh et al. (2023)	UK	Interventional Non‐RCT	28 days	F: 6 M: 3	18–65	Healthy	247 mL/day	Grain‐based kefir fermentation	Around 30 billion CFU
Yilmaz et al. (2019)	Turkey	RCT	4 weeks	F: 12 M: 13	19–68	IBD (CD, UC)	400 mL/day	N/R	2.0 × 10^10^ CFU/mL viable *Lactobacillus* bacteria: L. pentosus, *L. brevis*, *L. plantarum*, *L. fermentum*, *L. kefiri*, and *Lactobacillus lindneri*
Öneş et al. 2025	Turkey	RCT	28 days	F: 12 M: 0	18–29	Healthy (Athlete)	200 mL/day	N/R	N/R
Çıtar Dazıroğlu et al. 2024	Turkey	Interventional Non‐RCT	8 weeks	F: 17 M: 0	18–40	PCOS	250 mL/day	N/R	*Lactobacillus kefiranofaciens subsp. kefirgranum, L. kefiri, L. acidophilus, Lactobacillus parakefiri, Lactobacillus bulgaricus, L. reuteri, L. casei, L. fermentum, L. helveticus, Lactococcus lactis, Leuconostoc mesentereoides, Bifidobacterium bifidum, Streptococcus thermophilus, Kluyveromyces marxianus, Kluyveromyces lactis, Acetobacter pasteuri anus, and Saccharomyces cerevisiae*. *Lactobacillus spp*.: 10.54 log kob/mL; *Lactococcus spp*.: 10.62 log kob/mL; and yeast: 2.69 log kob/mL
Bellikci‐Koyu et al. (2019)	Turkey	RCT	12 weeks	F: 10 M: 2	18–65	Metabolic Syndrome	180 mL/day	Culture DC1500I; full‐fat milk	*Lactococcus lactis subsp. lactis, Lactococcus lactis subsp. cremoris, Lactococcus lactis subsp. diacetylactis, Leuconostoc mesenteroides subsp. cremoris, L. kefiri, Kluyveromyces marxianus, and Saccharomyces unisporus*
Gupta et al. (2024)	USA	Interventional Non‐RCT	11 days	F: 6 M: 7	> 18	Critically ill (ICU)	Escalating dose (60–240 mL/day)	Lifeway® kefir from grains	*Bifidobacterium lactis, Lactobacillus lactis, Saccharomyces florentinus, Streptococcus diacetylactis, L. acidophilus, B. Longum, L. casei, L. reuteri, L. plantarum, L. rhamnosus, Bifidobacterium bacterium breve, and Leuconostoc cremoris*.
Lee et al. (2021)	Taiwan	RCT (Crossover)	28 days	M: 16 F: 0	20–30	Healthy	20 g/day (SYNKEFIR)	Spray‐dried fermented milk with maltodextrin	*L. paracasei* DSM 32785 (LPC12), *L. rhamnosus* DSM 32786 (LRH10), *L. helveticus* DSM 32787 (LH43), *L. fermentum* DSM 32784 (LF26), and *S. thermophilus* DSM 32788 (ST30)

*Note:* F: Female, M: Male, PCOS: Polycystic ovarian syndrome, IBD: inflammatory bowel disease, UC: Ulcerative colitis, CD: Crohn disease, CFU: Colony forming unit, N/R: Not reported, *L. plantarum: Lactiplantibacillus plantarum*, *L. rhamnosus: Lacticaseibacillus rhamnosus*, *L. casei: Lacticaseibacillus casei*, *L. paracasei: Lacticaseibacillus paracasei*, *L. reuteri: Limosilactobacillus reuteri*, *L. fermentum: Limosilactobacillus fermentum*, *L. brevis: Levilactobacillus brevis, L. acidophilus: Lactobacillus acidophilus, L. helveticus: Lactobacillus helveticus, L. kefiri: Lactobacillus kefiri, B. Longum: Bifidobacterium Longum*.

**Table 2:** Gut microbiota outcomes following kefir intervention.

Study	Analysis method	Taxonomic changes (Phylum/Class/Genus/Species)	Alpha diversity (method/p)	Beta diversity (method/p)	Kefir strain colonization
Walsh et al. (2023)	Shotgun metagenomics	Species: *Lactococcus raffinolactis* ↑	No change Shannon (*p* = 0.43) Simpson (*p* = 0.29) diversity indices	No change Bray–Curtis dissimilarity (*p* = 0.99)	Yes
Gupta et al. (2024)	Shotgun metagenomics	Species: *L. plantarum*, *L. reuteri*, *L. rhamnosus* ↑; *B. longum, Hungatella hathewayi, Clostridium bolteae* ↓	Alpha diversity ↓ Shannon Index (*p* = 0.048) Species richness (*p* = 0.01)	Beta diversity ↓ Bray–Curtis (*p* = 0.004)	Yes
Öneş et al. (2025)	16S rRNA	Phylum: *Verrucomicrobia, Euryarchaeota* ↑; *Proteobacteria* ↓ Genus: *Akkermansia, Bifidobacterium* ↑; *Bacteroides, Faecalibacterium* ↓ Species: *A. muciniphila* ↑, *Roseburia faecis* ↑	No change Shannon diversity and Chao1 richness (*p* > 0.05)	No change Bray–Curtis Dissimilarity (*p* > 0.05)	NR
Çıtar Dazıroğlu et al. (2024)	16S rRNA	Phylum: *Firmicutes, Actinobacteria* ↑; *Bacteroidetes, Proteobacteria* ↓ Class: *Clostridia, Bacilli* ↑; *Bacteroidia* ↓ Genus: *Lactococcus* ↑, *Holdemania* ↓	No change Shannon, Simpson, and Chao1 (*p* > 0.05)	No change unweighted and weighted unifracs (*p* > 0.05)	NR
Bellikci‐Koyu et al. (2019)	16S rRNA	Phylum: *Firmicutes*, *Actinobacteria* ↑; *Bacteroidetes* ↓ Class: *Clostridia* ↑ Genus: *Veillonellaceae* ↓, *Bacteroides* ↑	No change Faith's Phylogenetic Diversity, Pielou's Evenness, and Shannon Index (*p* > 0.05)	No change Jaccard index, Bray–Curtis Dissimilarity, and unweighted and weighted unifracs (*p* > 0.05)	Yes
Choi et al. (2025)	16S rRNA	Phylum: *Firmicutes* ↑, *Bacteroidetes* ↓ Genus: *Bifidobacterium, Blautia* ↑ Species: *Blautia wexlerae, Blautia luti, Lactococcus lactis, Bifidobacterium breve, Ruthenibacterium lactatiformans, Weissella koreensis, and Leuconostoc mesenteroides* ↑ *S. thermophilus* ↓	No change Chao1, Shannon, and Simpson (*p* > 0.05)	No change weighted and unweighted UniFrac distances (*p* > 0.05)	Yes
Lee et al. (2021)	qPCR	Genus: *Bifidobacterium* ↓	NR	NR	N/R
Yilmaz et al. (2019)	qPCR	Strain: *L. kefiri*	NR	NR	Yes

*Note:* rRNA: Ribosmal RNA, qPCR: Quantitative polymerase chain reaction, N/R: Not reported, N/A: Not applicable, *L. plantarum*: *Lactiplantibacillus plantarum*, *L. rhamnosus*: *Lacticaseibacillus rhamnosus*, *L. reuteri*: *Limosilactobacillus reuteri*, *L. kefiri*: *Lactobacillus kefiri*, *B. Longum*: *Bifidobacterium Longum*.

### 2.4 Quality Assessment

All studies selected for retrieval were assessed by two independent reviewers (M.H. and Y.R.). The quality of the included randomized controlled trials was evaluated according to Cochrane risk‐of‐bias tool for randomized trials (RoB 2) (Higgins 2011). This tool has the following key parts: (1) random sequence generation, (2) allocation concealment, (3) blinding of participants and personnel, (4) blinding of outcome assessment, (5) incomplete outcome data, (6) selective reporting, (7) and other bias (other sources of bias that have been detected by the reviewer). Each item was categorized as having a low/unclear/high risk of bias. Accordingly, studies with more than two items of low risk were categorized as studies with good quality, studies with two items of low risk were considered studies with fair qualities, and with fewer than two items with low risk of bias, they were considered studies with weak qualities. Risk of Bias of Non‐Randomized Trials (ROBINS‐1) was used to assess the risk of bias of non‐randomized trials (Sterne 2016).

## 3 Results

**Figure 1:**
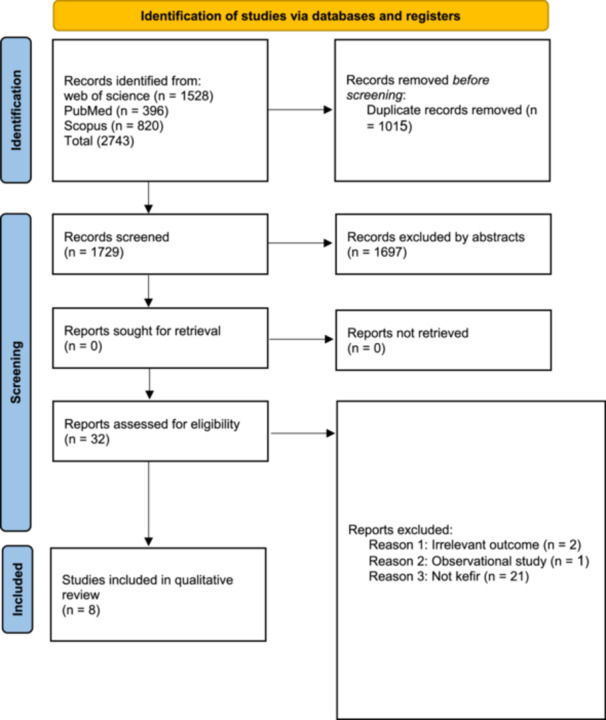
Prisma flow diagram.

A total of 2743 relevant papers (Web of Science: 1527, PubMed: 396, and Scopus: 820) were included after excluding 1015 duplicate studies (Figure 1). By screening the title and abstract, 1697 ineligible studies were further ruled out. The remaining 32 articles were obtained and screened for further evaluation. Ultimately, eight studies that met the inclusion criteria were included in this systematic review.

### 3.1 Studies’ Characteristics

Eight studies involving 117 participants were included in the review, comprising healthy individuals (Walsh et al. 2023; Choi et al. 2025), patients with irritable bowel disease (IBD) (Yilmaz et al. 2019), athletes (Lee et al. 2021; Öneş et al. 2025), women with PCOS (Çıtar Dazıroğlu et al. 2024), individuals with metabolic syndrome (Bellikci‐Koyu et al. 2019), and critically ill patients (Gupta et al. 2024). The duration of studies ranged from 11 days to 12 weeks. The studies were performed in various geographical locations including Turkey (Yilmaz et al. 2019; Öneş et al. 2025; Çıtar Dazıroğlu et al. 2024; Bellikci‐Koyu et al. 2019), Tiwan (Lee et al. 2021), USA (Gupta et al. 2024), Korea (Choi et al. 2025), and UK (Walsh et al. 2023). Out of 8 studies, two studies were exclusive to females (Öneş et al. 2025; Çıtar Dazıroğlu et al. 2024), one study was exclusive to males (Lee et al. 2021) (Table 1). Most included studies used kefir in its liquid form with doses varying between 150 and 400 mL/day (Walsh et al. 2023; Choi et al. 2025; Yilmaz et al. 2019; Öneş et al. 2025; Çıtar Dazıroğlu et al. 2024; Bellikci‐Koyu et al. 2019; Gupta et al. 2024), and one of them provided SYNKEFIR product (Lee et al. 2021). Further details regarding kefir preparation method and kefir starter microbial content are provided in (Table 2).

### 3.2 Risk of Bias Assessment

**Figure 2:**
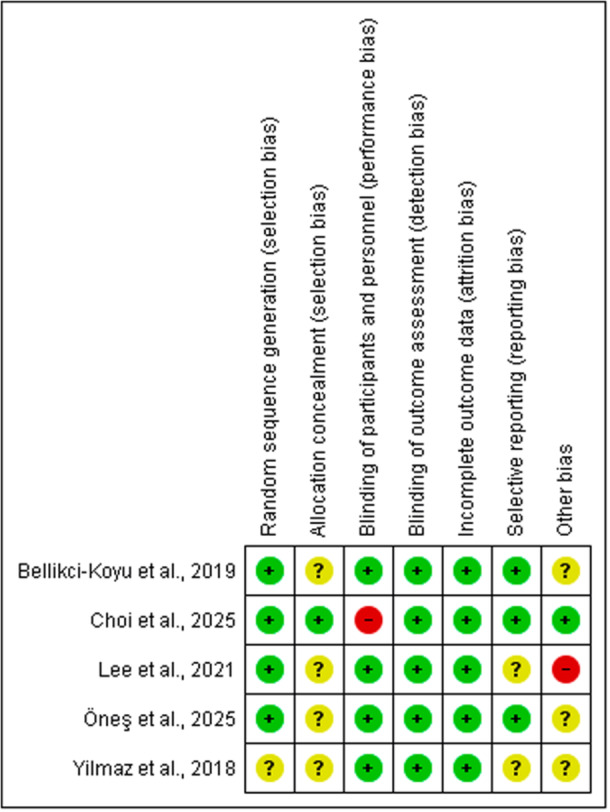
Risk of bias summary of included RCTs.

**Figure 3:**
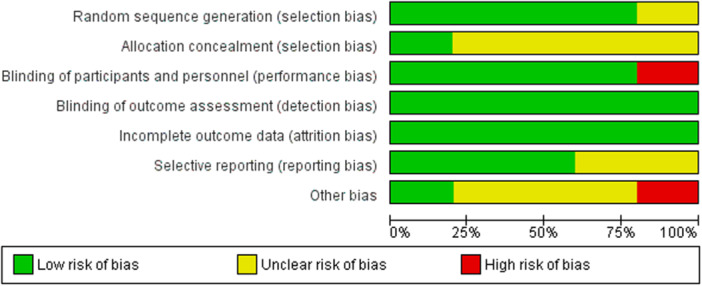
Risk of bias graph of included RCTs.

**Figure 4:**
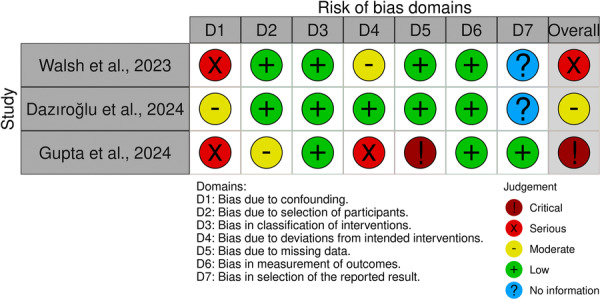
Risk of bias summary of included non‐RCTs.

Risk‐of‐bias assessment was performed in all the included studies. The assessment of RCTs revealed that all of the studies were at low risk of bias (high quality). Risk of bias summary is presented in **(**Figure 2 **)** and the graph is shown in **(**Figure 3 **)**. It should be noted that studies with unclear description of kefir's microbial content were assessed as unclear in the other bias domain. Similarly, studies with kefir lacks yeasts in its content were assessed as high risk in the other bias domain. Non RCTs assessment revealed that 1 of the studies had critical risk, 1 of the studies had serious risk, and 1 of the studies had moderate risk (Figure 4).

### 3.3 Microbiome Quantification

Two studies used qPCR (Yilmaz et al. 2019; Lee et al. 2021). Three studies used 16S rRNA sequencing (Choi et al. 2025; Öneş et al. 2025; Çıtar Dazıroğlu et al. 2024; Bellikci‐Koyu et al. 2019), and two studies performed shotgun metagenomic sequencing (Walsh et al. 2023; Gupta et al. 2024) (Table 2).

### 3.4 Effect of Kefir Consumption on Gut Microbiota Composition

Consumption of 180 mL/day of kefir for 3 months in 12 patients with metabolic syndrome was investigated using the 16S rRNA technique to assess gut microbial change. This study resulted in a mild increase in *Firmicutes* (30% vs. 39%), decrease in *Bacteroidetes* (51% vs. 39%), and a significant increase in the relative abundance of the phylum *actinobacteria* (0.003% vs. 0.03%) at the end of the study. In addition, there were slight increases in *Clostridia* (73% vs. 85%), *Lactobacillales* (2% vs. 5%), (31% vs. 39%), and a decrease in *Veillonellaceae* (9% vs. 6%). It should be noted that *bifidobacterium* detection was in 50% of the participants at the beginning of the study, whereas 91.7% at the end of the study. On the other hand, *Verrucomicrobia* was detected in 75% at the beginning of the study and 58.3% at the end of the study. In addition, there was no change in alpha and beta diversity during the study period (Bellikci‐Koyu et al. 2019).

Furthermore, in 17 women with PCOS, consumption of 250 ml/day for 2 months revealed slight decreases in *Bacteroidetes* (28% vs. 23.8%), *proteobacteria* (2.9% vs. 1.8%), and increases in *Firmicutes* (66% vs. 69%) and *actinobacteria* (1.8% vs. 3.6%). On the class level, there was an increase in *clostridia* (63.2% vs. 65.8%), *Actinobacteria* (1% vs. 2.8%), and *Bacilli* (0.8% vs. 1.8%), and a decrease in *Bacteroidia* (28% vs. 23.6%), *Gammaproteobacteria* (2.3% vs. 1%), and *Erysipelotrichia* (*p* = 0.11). Only *Bacilli* change was reported to be statistically significant (*p* = 0.04). This increase in *Bacilli* class has been reported to be from *lactooccocus* genus (*p* = 0.09) and the decrease in *Holdemania* genus (*p* < 0.05) from *Erysipelotrichia* class. No difference in alpha and beta diversity was observed at the end of the study (Çıtar Dazıroğlu et al. 2024).

In professional female football players, there was an increase in phyla *Verrucomicrobia* (1,9% vs 3.3%) and *Euryarchaeota* (1.4% vs 3%) and decrease in *Proteobacteria* (2.6% vs. 2.4%). The relative abundance of some genera, including *Bacteroides* (15.7% vs. 13.5%) and *Faecalibacterium* (14.8% vs. 13.6%) was decreased, and the relative abundances of potentially healthy bacteria, including *Akkermansia* (2.4% vs. 4.1%) and *Bifidobacterium* (2.4% vs. 4.1%) was increased. Interestingly, *Faecalibacterium prausnitzii (F. Prausnitzii)* was more abundant in high performance group compared to the low performance group, potentially due to its energy production role. Moreover, on the species level, a statistically significant increase in *A. muciniphila* (4.1% vs. 8.5%) was reported. Considerable increase in some species including *Roseburia faecis* (*R. faecis*) (6.4% vs. 7.5%), *Roseburia inulinivorans* (1.2% vs. 2.2%), and *Blautia obeum* (0.8% vs. 1.5%), and decrease in *Ruminococcus bromii* (6.7% vs. 4.7%), and *Bacteroides ovatus* (1,8% vs 0.4%) following kefir consumption. No effect was observed *Firmicutes* to *Bacteroidetes* ratio. Alpha and beta diversity didn't differ between before and after study (Öneş et al. 2025). Another study examined the effect of 20 grams/day of kefir powder (SYNKEFIR) on endurance performance for 8 weeks. Using qPCR method, four major genera were measured which included *bifidobacterium*, *lactobacillus*, *Bacteroides* and *clostridium*. A significant reduction in *bifidobacterium* was found after SYNKEFIR intervention, and no other difference was reported (Lee et al. 2021).

In an RCT involving patients with IBD, 400 mL/day of kefir was consumed daily for 1 month. qPCR analysis demonstrated an increase in the relative abundance of *lactobacillus* in fecal samples following the intervention, largely driven by the detection of *lactobacillus kefir* (*L. kefiri*) strain (Yilmaz et al. 2019).

Moreover, Walsh et al. (2023) has compared the efficacy of inulin, fermented milk products, and kefir on gut microbial composition using shotgun metagenomic sequencing. Alpha and beta diversity were not changed by any of the interventions. Only kefir consumption was associated with an increased relative abundance of *Lactococcus raffinolactis* (*Lc. Raffinolactis*) in 4 of 9 participants at the end of the study, indicating post‐intervention detection of a kefir‐associated species (Walsh et al. 2023)

A recent RCT on healthy young adults investigated various milk products including milk, yogurt, and kefir on gut microbiota composition for 2 weeks. Despite the short duration of intervention, the study was designed to minimize the effect of dietary and environmental variability through providing the same diet to the participants along with staying at job experience camp. Aligning with the other studies, there was no effect observed in alpha and beta diversity. Compared to pre intervention, there was an increase in the phyla *Firmicutes* (67.73% to 71.46%) and a decrease in *Bacteroidetes* (23.45% to 17.32%. At the genus level, the levels of *Bifidobacterium* and *Blautia* rose from (6.87% to 9.92% and (9.17%−13.98%) respectively. Within the genus *Blautia*, the species *Blautia wexlerae and Blautia luti* were increased compared to baseline. In addition, several lactose fermenting bacterial species including *Bifidobacterium breve, Ruthenibacterium lactatiformans, Weissella koreensis*, and *Leuconostoc mesenteroides* were significantly increased after the intervention. In addition, *Lactococcus lactis* which is one the species that make up the kefir grains, exhibited a significant increase compared to baseline. These effects were not observed in the other interventions (Choi et al. 2025).

A study was conducted on critically ill patients in which gut microbial composition was assessed at ICU admission and after 72 h. Kefir was provided to patients in escalating doses starting by 60 mL, 120 mL after 12 h, and 240 mL daily. Stool samples of two time points were collected from 13 patients. The patients experienced a reduction in overall count of gut microbial phyla and genus between first measurement (T1) and second measurement (T2). Alpha diversity analysis confirmed those findings in which a significant decrease in Shannon index (*p* = 0.04) and species richness (*p* = 0.01) was found. Similarly, beta diversity was significantly reduced (*p* = 0.004). The results were justified by the nature of ICU medical settings in which patients had to administer antibiotics. The most prevalent phyla were *Firmicutes* and *Bacteroidetes*, and *bacilli* and *bacteroidia* were the most prevalent classes. In addition, The Gut Microbiome Wellness Index (GMWI) was used to compare the change in the relative abundance of each GMWI species. There was a significant increase in GMWI between T1 and T2 (*p* = 0.03). Out of 42 species, only 23 species were detected in patients gut microbiota samples; 22 were disease‐associated species, of which 16 were non‐significantly reduced. Moreover, species *Hungatella hathewayi* and *Clostridium boltea* were significantly decreased compared to baseline (*p* < 0.05). In contrast, *Parvimonas micra* and *Dialister pneumosintes* disease‐associated species were slightly increased. The authors further assessed the presence of Kefir 12 bacterial species in patients gut between T1 and T2. Out of 12 species, only 5 were detected: *Bifidobacterium longum (B. Longum), Lactiplantibacillus plantarum (L. plantarum)*, *Limosilactobacillus reuteri (L. reuteri)*, *Lactobacillus acidophilus (L. acidophilus)*, and *Lacticaseibacillus rhamnosus (L. rhamnosus)*, at T1 and T2. *L. plantarum*, *L. reuteri*, and *L. acidophilus* were increased, whereas *B. Longum* was decreased at the end of the study (Gupta et al. 2024).

## 4 Discussion

To the best of our knowledge, this is the first systematic review to assess kefir consumption's impact on gut microbiota composition. This review included 8 studies comprising of different populations, health conditions, and kefir microbial content. Our findings reveal that kefir consumption has led to some shifts in the gut bacterial phyla, represented by increase in *Firmicutes*, *Actinobacteria*, and decrease in *Bacteroidetes* (Choi et al. 2025; Çıtar Dazıroğlu et al. 2024; Bellikci‐Koyu et al. 2019) and *Proteobacteria* (Choi et al. 2025; Çıtar Dazıroğlu et al. 2024). The ratio of *Firmicutes* to *Bacteroidetes* was not altered (Öneş et al. 2025; Bellikci‐Koyu et al. 2019). A rise in the classes *Clostridia* (Çıtar Dazıroğlu et al. 2024; Bellikci‐Koyu et al. 2019), *Bacilli*, and *Actinobacteria* was observed (Çıtar Dazıroğlu et al. 2024). Health‐promoting genera *Bifidobacterium* (Choi et al. 2025; Öneş et al. 2025; Bellikci‐Koyu et al. 2019) and *Akkermansia* were increased, whereas *Bacteroides and Faecalibacterium* were reduced (Öneş et al. 2025). In contrast, Lee et al. (2021) found a significant reduction of *Bifidobacterium* when compared to placebo (Lee et al. 2021). Moreover, *F. prausnitzii* was more abundant in high performance group compared to the low performance group. Furthermore, Bellikci‐Koyu et al. (2019) reported an increase in *Lactobacillales* order from 2% to 5% during the study (Bellikci‐Koyu et al. 2019). On the species level, shift toward healthy microbial composition was reported by Gupta et al. (2024) in which an increase in *A. muciniphila, R. faecis*, *Roseburia inulinivorans*, and *Blautia obeum*, and a decrease in *Ruminococcus bromii*, and *Bacteroides ovatus* was found (Gupta et al. 2024). In addition, some kefir comprising species could have the ability to proliferate in the human colon; Gupta et al. (2024) found an increase in 4 *lactobacillus* species in fecal samples, namely, *L. plantarum*, *L. reuteri*, *L. acidophilus*, and *L. rhamnosus (*Gupta et al. 2024 *)*. Similarly, Walsh et al. (2023) found *Lc. Raffinolactis* in 4 out of 9 participants due to kefir consumption (Walsh et al. 2023). Choi et al. (2025) reported a significant increase of *Lactococcus lactis* compared to baseline (Choi et al. 2025). Yilmaz et al. (2019) reported an increase in *L. kefiri* strain, the main microbe of kefir grains, in fecal samples of IBD patients at the end of the study (Yilmaz et al. 2019).

Dysbiosis is a condition characterized by an imbalance between beneficial and harmful gut microorganisms. It is linked to various health diseases such as obesity, low grade inflammation, and metabolic syndrome (Alagiakrishnan et al. 2024). The changes induced by kefir consumption highlight its potential role in shifting gut microbiota composition towards healthier in humans, which may result in eubiosis. Several in vivo studies investigated the impact of kefir on gut microbiota and reported positive outcomes overall. A study on dogs reveals that kefir consumption increased lactic acid producing bacteria (LAB) species and *F. prausnitzii* along with reduction in *Enterobacteriaceae (*Kim et al. 2019 *)*. In rats, kefir administration for 5 weeks has increased *Bacteroidetes*, *yeasts*, *lactobacillus*, and *Lactococcus*, and decreased *Proteobacteria*, *Firmicutes*, and *Enterobacteriaceae*. The LAB: *Enterobacteracae* remained elevated even after 1 week washout which indicates successful inhibition of *Enterobacteriaceae* growth (Kim et al. 2015). Similarly, doses of 10, 20, and 30 mL/kg of kefir, compared to control, resulted in increased *lactobacillus* and yeasts, and decreased *Enterobacteria*. The impact of kefir was dose‐dependent in which higher doses (e.g. 30 mL/kg) exerted better improvements. Interestingly, 30 mL/kg has significantly inhibited pathogenic *Enterobacteria* compared to other doses and control groups (Ozsoy et al. 2021)).

One of the important aspects regarding changes in gut microbiota composition is the ratio of *Firmicutes* to *Bacteroidetes*. While higher ratios are associated with chronic diseases such as obesity, metabolic syndrome, and fatty liver disease, lower ratios are associated with improved metabolic health. The exact role of these microorganisms remains unclear (An et al. 2023; Magne 2020). Previously, it was suggested that elevated ratio of *Firmicutes* to *Bacteroidetes* are associated with higher BMI due to their energy harvesting properties in host intestine (Turnbaugh et al. 2006). However, current studies failed to show any correlation, specifically in humans (Houtman et al. 2022; Karačić et al. 2024). A recent analysis of IBD patients found higher *Bacteroidetes* than *Firmicutes* (lower ratio of *Firmicutes* to *Bacteroidetes*) in patients with active disease. In addition, shift of microbiota toward increase in *Firmicutes* to *Bacteroidetes* was found to improve the disease state (Tsai et al. 2025). In the current review, kefir failed to produce significant effect on *Firmicutes* to *Bacteroidetes*, despite having minor increase on *Firmicutes* and reduction on *Bacteroidetes* phyla. The observed increase in the *Firmicutes* phylum following kefir consumption may be attributed to the proliferation of *Lactobacillus* species and other related genera within the class *Bacilli*, which were commonly increased in included trials.

### 4.1 Possible Mechanisms

Potential mechanistic pathways of how kefir beverage could exert its effect on gut microbiota include: colonization of beneficial bacteria (1) modulation of colon environment resulting in increase of beneficial and reduction of pathogenic bacteria (2), and production of antimicrobial metabolites (3). Further explanation of these mechanisms is provided below.

### 4.2 Colonization of Kefir Bacteria in Gut

Food‐borne microbial colonization in the human colon has been discussed previously, and the ability of colonization occurrence is yet to discover (Roselli 2021). Gut microbiotas form a stable microbial ecosystem that resists foreign invaders, which was well established with respect to pathogenic bacterial species such as *Escherichia coli (E. coli)* and *Clostridium difficile* (*C. difficile*) (Pickard and Núñez 2019); Wilson et al. 1986). However, recent evidence suggests microbial colonization depends on existing gut microbiota composition which was confirmed to be heterogenous among individuals. While some may initially have ‘resistant’ and the other ‘permissive’ gut microbial community, both may exert different dynamics toward transient microbes (e.g. food‐borne bacteria) (Zhang et al. 2016). For instance, the daily average dose consumption of *L. lactis* isolated from fermented milk products (FMP) was more rapidly eliminated in resistant group compared to permissive group by a difference of approximately 2 days in rats. In addition, relative abundance of some taxa were changed after FMP consumption. However, the baseline values of these taxa were recovered within 2 days in resistant, but not permissive, group indicating lower resilience of permissive group. The same authors repeated the experiment on human participants; they found reduction in *Lactococcus* from 100% (during FMP consumption period) to 36% (during wash out period) (Zhang et al. 2016). Moreover, colonization ability of *L. kefiri* LKF01 isolated from kefir grains was examined in 20 healthy subjects. Supplementation for 1 month resulted in *L. kefiri* LKF01 detection in all the participants at the end of the study. Interestingly, 1 month after the end of the study, *L. kefiri* LKF01 was recovered only in three subjects (Toscano et al. 2017). These findings suggest that baseline gut microbial configuration may influence the transient persistence of exogenous microbes.

In the current review, four studies reported increased detection of kefir‐associated taxa in fecal samples after consumption. *Bifidobacterium* detection was noticed in additional 40% of total participants after kefir consumption (Bellikci‐Koyu et al. 2019) The species *Lactococcus lactis* which comprised 6.2% of the kefir grains showed a significant increase compared to baseline (Choi et al. 2025) Yilmaz et al. (2019) detected *L. kefiri* strains in fecal samples in 7 out of 10 IBD patients’ feces (Yilmaz et al. 2019). Interestingly, Gupta et al. (2024) detected four kefir induced species including *L. plantarum*, *L. reuteri*, *L. acidophilus*, and *L. rhamnosus* in critically ill patients' feces after acute kefir consumption (Gupta et al. 2024). The higher detection of bacterial species in Gupta et al. (2024) study compared to the other included human trials could be explained by antibiotic administration, resulting in lower competition between commensal and transient gut microbiota. It was demonstrated that probiotic supplementation after antibiotic course yielded to the detection of product strains in humans and rats, which could be due to lower competition among gut community thus alleviating the colonization of microbes (Suez et al. 2018; Theriot et al. 2022) In contrast, a recent meta‐analysis concluded that probiotic supplementations during or after antibiotic course is unjustified in humans (Éliás et al. 2023) Although findings from Gupta et al. (2024) confirm bacterial colonization after antibiotic course, further studies examining the role of probiotic consumption after antibiotic course are critical to understand the possibility of colonization.

Moreover, a study by Walsh et al. (2023), *Lc. Raffinolactis*, which is not one of the dominant bacteria in kefir, was detected in 4 out of 9 healthy subjects (Walsh et al. 2023). The authors further analyzed the genetic material to find associated genes of *Lc. Raffinolactis* that might play a role in colonization properties. A total of 25 genes associated with host adaptability were identified. Twenty‐three of them were found to be survival genes which provide defense properties such as tolerating various GI tract conditions and reaching colon. The other three genes were associated with colonization effects indicating direct interaction between *Lc. Raffinolactis* and mucus layer. Among them, carbohydrate activated enzymes GH109 and GH42 are related with mucin cleavage, while the peptidase Sortase A‐C60A is associated with mucin binding ability, which collectively provide colonization properties (Walsh et al. 2023).

### 4.3 Modulating the Environment of Colon

The healthy colon is characterized by low oxygen availability, and high relative abundance of obligate anaerobes, mainly including *Firmicutes* and *Bacteroidetes* (Rigottier‐Gois 2013; Van Hul et al. 2024). Colon environment is usually disrupted in chronic diseases such as PCOS and IBD, thus leading to increased oxygen availability, reduced acidity, and reduced obligate anaerobic bacteria (Rigottier‐Gois 2013; Li et al. 2025). This shift on colon environment leads to increase in the abundance of harmful bacteria and reduces beneficial bacteria and their metabolites (Rigottier‐Gois 2013; Li et al. 2025; Gautam et al. 2024). Short chain fatty acids (SCFAs) are among the main bacterial metabolites, and their abundance is associated with healthy gut (Silva et al. 2020; Fusco 2023). In current research, kefir intake has been shown to increase the abundance of SCFA producing bacteria levels such as *Lactobacillus, Lactococcus, Bifidobacterium, and R. faecis (*Walsh et al. 2023; Yilmaz et al. 2019; Lee et al. 2021; Öneş et al. 2025; Çıtar Dazıroğlu et al. 2024; Bellikci‐Koyu et al. 2019; Gupta et al. 2024). Out of the included studies, only Walsh et al. (2023) directly quantified SCFA changes during the study period. The authors found significant increase in acetic acid, succinic acid, Betaine, and N,N‐Dimethylglycine which their annotations were also found in *Lc. Raffinolactis* genes (Walsh et al. 2023).

Moreover, it was noted that the production of SCFA, such as lactic acid by LAB, shifts colon environment into more acidic and anaerobic and subsequently supresses pathogen proliferation such as *Enterobacteriaceae* along with increase in beneficial bacteria (Den Besten et al. 2013). Similarly, included studies have found reduction in harmful bacteria like *Veillonellaceae, Gammaproteobacteria, proteobacteria, Bacteroidia, Hungatella hathewayi and Clostridium boltea* and increase in beneficial bacteria *lactobacillus, Bifidobacterium, lactoccocus, Akkermansia*. Furthermore, the decline in pH (5,5) values in the colon was associated with higher butyrate producing bacteria such as r*oseburia* spp. and *F. prausnitzii* in human feces. However, acetate‐ and propionate producing bacteria become more dominant and butyrate producing bacteria disappears when pH increases (6.5) (Den Besten et al. 2013). Notably, Öneş et al. (2025) reported increase in *R. faecis* and *Roseburia inulinivorans* and found positive correlation between *F. prausnitzii* abundance and exercise performance (Öneş et al. 2025). Butyrate plays a critical role in maintaining anaerobic condition of colon by facelifting its usage by colonocytes which increases their oxygen utilization (Singh 2023). Also, SCFA may directly act as a substrate for other beneficial gut microbes, thus aiding to nourish them and increase their relative abundance through cross feeding mechanisms (Ríos‐Covián et al. 2016). Therefore, modulation of colon environment plays a significant role in increasing beneficial (e.g. *lactobacillus*) and decreasing pathogenic bacteria (e.g. *enterobacteria*).

### 4.4 Production of Antimicrobial Agents

Eliminating harmful bacteria through colon environment modulation is not the only function of SCFA. Further, intense growth competition in the colon has led the bacteria to develop some weapons to suppress or kill invaders (Pickard and Núñez 2019). SCFA, bacteriocins, and exopolysaccharides are all bacterial metabolites, specifically LAB and acetic acid bacteria, carrying antimicrobial properties. Several reviews have discussed this topic in detail (Anjana and Tiwari 2022; Pickard and Núñez 2019; González‐Orozco et al. 2022). Briefly, LAB isolated from kefir produces bacteriocins which may be responsible for the anti‐microbial action in a wide range of bacterial strains. Isolated bacteriocin F1 from *Lacticaseibacillus paracasei* subsp. of kefir has shown to inhibit both bacteria and fungi, such as *E. coli*, *Staphylococcus aureus*, *Bacillus thuringiensis*, *Salmonella enterica*, *Shigella dysenteriae*, *Aspergillus flavus*, *Aspergillus niger*, *Rhizopus nigricans*, and *Penicillium glaucum (*Wannun et al. 2014 *)* In addition, LAB bacteriocins exerted inhibitory effects against foodborne pathogens such as *Listeria monocytogenes* and several *enterococci* present in human intestine. *Pediococcus acidilactici* UL5 produces pediocin PA‐1, which showed anti‐listerial activity in a mouse model without affecting the native intestinal microbiota (Anjana and Tiwari 2022). Similarly, EPS kefiran has also exerted inhibitory effects against *Streptococcus pyogenes*, *Streptococcus salivarius, Salmonella Typhimurium, Candida albicans*, and *Listeria monocytogenes (*Rodrigues et al. 2005 *) L. kefiranofaciens DN1* metabolite EPS_DN1 inhibited the growth of *Listeria monocytogenes* and *Salmonella enteritidis* (Jeong et al. 2017). Moreover, denoted FK‐1000 fraction isolated from kefir has exerted inhibitory effects on some weak‐acid resistant microbes such as *P. aeruginosa* and *Methicillin‐Resistant Staphylococcus Aureus*, pathogens associated with Nosocomial infections, at 7 pH (Marques et al. 2020). Collectively, the antimicrobial agents synthesized by kefir‐derived bacterial strains and yeast suppress pathogenic bacteria, which could partially explain our results.

### 4.5 Strengths, Limitations and Future Directions

While this systematic review provides novel insights into the effects of kefir consumption on human gut microbiota, several limitations should be acknowledged. First, the number of included studies (*N* = 8) and participants (*N* = 117) was relatively small, which limits the strength of the conclusions. The lack of sufficient statistical data (e.g., mean and standard deviation for pre‐ and post‐intervention outcomes) across included studies limited multiple comparison across many taxa and prevented us from performing a quantitative synthesis through meta‐analysis. In addition, the included studies were conducted across different ethnicities, geographical locations, and health conditions, all of which are known to influence gut microbiota composition and may lead to heterogeneous outcomes. Sex differences may also affect gut microbiota, as two studies included only female participants and one study consisted of males. Furthermore, it remains unclear whether the observed microbiota changes are sufficient to produce meaningful health benefits in already healthy populations. Although only interventional studies were included, substantial heterogeneity existed beyond intervention characteristics such as kefir dose, composition, duration of consumption, and microbiota analytical methods. Differences in baseline health status and gut microbiota composition as well as unassessed lifestyle factors may have further contributed to variability in the findings.

Furthermore, commonly used diversity metrics such as alpha and beta diversity reflect overall microbial community structure and may not detect minor changes in specific taxa. In this case, kefir intake may increase beneficial genera such as *Lactobacillus* or *Bifidobacterium* without substantially altering overall richness or evenness of the gut microbiota. While *lactobacillus* measurement was common in included studies, some studies specified their analysis to certain bacterial phyla, genus, species, or strains, making the comparison challenging. All the studies used fecal samples to measure the relative abundance of bacteria. Despite that fecal samples are commonly used in literature to assess gut microbial composition; their results are limited to approximate colon microbiota. Therefore, changes in upper gastrointestinal tract such as stomach, and small intestines have not been measured yet. While all studies reported changes in gut microbiota composition following kefir consumption, there remains a notable lack of functional readouts that could help determine the biological relevance of these microbial shifts. For example, only few studies assessed downstream functional outcomes such as microbial metabolite production (e.g. SCFA), which limits our ability to interpret whether the observed compositional changes translate into meaningful physiological effects.

Finally, the quality of included trials was mostly low to medium, mainly due to potential selection bias and confounding factors such as physical activity and uncontrolled dietary intake. An important confounding factor in such interventional trials is the matrix of kefir beverage. Separation of the effects caused by kefir probiotics or kefir nutritional components (e.g. vitamins and minerals) in humans is impossible due to their potential synergistic effects. Collectively, this clinical and methodological heterogeneity limits direct comparability between studies and reduces the generalizability of the results, highlighting the need for large scale well controlled RCTs. Future studies should use standardized kefir formula with clear description of bacterial and yeast content on strain level as well as their viability. In addition, incorporation of functional microbiome assessment such as metabolite production (e.g. SCFA) and host metabolic markers (e.g. gut barrier permeability) is critical to determine whether observed shifts translate into meaningful physiological benefits. Consideration of these points will improve the comparability of kefir consumption induced effects on gut microbiome across trials.

## 5 Conclusion

Kefir consumption was associated with modest and heterogenous changes in gut microbiota composition across the included human intervention studies. Proposed mechanisms may include transient persistence of kefir‐associated taxa, modulation of the intestinal environment and microbial metabolite production. However, this review has several limitations. The number of included studies and participants was relatively small, and the populations studied were highly heterogeneous in terms of ethnicity, health status, and sex, all of which are known determinants of gut microbiota composition. In addition, intervention‐related heterogeneity, such as the lack of standardized kefir formulations and the limited description of bacterial and yeast strains and their viability, may further limit the comparability of findings across studies. Commonly used diversity metrics, such as alpha and beta diversity, which were frequently reported in the included studies, may also be insufficiently sensitive to detect subtle microbial shifts induced by kefir consumption. Moreover, the limited assessment of microbiome functional outcomes, including microbial metabolite production and their interactions with host metabolic markers, further restricts the interpretation of the observed changes. Therefore, future studies should address these limitations to better clarify the relationship between kefir consumption and gut microbiota dynamics.

## Author Contributions

**Mohammed Hamsho:** conceptualization, data curation, formal analysis, visualization, writing – original draft, methodology, investigation, software, validation. **Yazan Ranneh:** project administration, supervision, writing – review and editing. **Batoul Kaddour:** data curation, formal analysis, investigation, methodology. **Sundus Alfakhri:** data curation, formal analysis, investigation, methodology. **Hale Hacıbayram:** data curation, formal analysis, methodology, investigation. **Wijdan Shkorfu:** data curation, formal analysis, methodology, investigation. **Merve Terzi:** data curation, formal analysis, writing – review and editing, project administration, supervision, investigation. **Abdulmannan Fadel:** data curation, formal analysis, supervision, project administration, writing – review and editing, investigation.

## Funding

The authors have nothing to report.

## Ethics Statement

The authors have nothing to report.

## Conflicts of Interest

The authors declare no conflicts of interest.

## Supporting information

Supporting File

## Data Availability

The data that supports the findings of this study are available in the supplementary material of this article.
