# Supplementary material for: Kefir: A Potential Gut Microbiota Modulator: A Systematic Review of Human Interventional Studies
Source: Microbiologyopen. 2026 Apr 26;15(3):e70297. doi: 10.1002/mbo3.70297 (PMC13111804; doi:10.1002/mbo3.70297)
Supplement: Supplementary file 1 — Supporting File [file MBO3-15-e70297-s001.docx]

**Table S1: Electronic search strategies**

| **Database** | **Search Strategy** |
| --- | --- |
| **PubMed** | 1. ("Kefir"[MeSH] OR kefir[tiab] OR "fermented milk"[tiab]) 2. AND ("Gastrointestinal Microbiome"[MeSH] OR "Intestinal Microbiota"[MeSH] OR "gut microbiota"[tiab] OR "gut microflora"[tiab] OR "intestinal microbiota"[tiab]) 3. AND ("Clinical Trial"[Publication Type] OR "Randomized Controlled Trial"[Publication Type] OR intervention[tiab] OR "interventional study"[tiab]) 4. AND (humans[MeSH Terms]) |
| **Scopus** | 1. TITLE-ABS-KEY(kefir OR "fermented milk") 2. AND TITLE-ABS-KEY("gut microbiota" OR "gut microflora" OR "intestinal microbiota" OR "gastrointestinal microbiota") 3. AND TITLE-ABS-KEY("clinical trial" OR "randomized controlled trial" OR intervention OR "interventional study") 4. AND TITLE-ABS-KEY(human OR humans OR adult OR participants) |
| **Web of Science** | 1. TS=(kefir OR "fermented milk") 2. AND TS=("gut microbiota" OR "gut microflora" OR "intestinal microbiota" OR "gastrointestinal microbiota") 3. AND TS=("clinical trial" OR "randomized controlled trial" OR intervention OR "interventional study") 4. AND TS=(human OR humans OR adult) |
